# Nursing strategies for severe poststroke fatigue: a case report of a structured, multidimensional intervention program

**DOI:** 10.3389/fresc.2026.1887052

**Published:** 2026-06-29

**Authors:** Qinqin Hu, Anna Wang, Qin Zhou, Junxian Huang, Haiyan Zhou

**Affiliations:** 1 Department of Neurology, Anqing Municipal Hospital, Anqing, China; 2 Department of Nursing, Anqing Municipal Hospital, Anqing, China

**Keywords:** case report, energy metabolism, poststroke fatigue, psychosocial support, stroke

## Abstract

Poststroke fatigue (PSF) is a complex, multidimensional symptom characterized by a persistent, disproportionate sense of exhaustion that is not fully relieved by rest. It typically manifests as early-onset fatigue with reduced energy reserves, significantly hindering post-stroke recovery. This article reports the nursing process of a patient with a right basal ganglia infarction complicated by severe PSF. After admission, the patient received intravenous thrombolysis and antiplatelet therapy, combined with early-stage rehabilitation. A personalized, phased nursing plan centered on “precise assessment-energy restructuring-sleep optimization-psychological empowerment-family collaboration” was implemented. Specific interventions included structured activity and rest diary management, personalized energy conservation technique training, sleep hygiene interventions, goal setting and behavioral activation using Motivational Interviewing (MI) techniques, and systematic education and support for caregivers. Following a 6-week intervention period, the patient was discharged. Follow-ups at 1, 3, and 6 months post-discharge indicated recovery.

## Introduction

Poststroke fatigue (PSF) is characterized by a persistent, disproportionate sense of exhaustion that is not alleviated by rest and significantly impairs daily function. Importantly, it is independent of both depressive symptoms and the severity of neurological deficits (1, 2). In the global context, international literature reports that the prevalence of PSF following stroke is 46.79% (3). PSF, independent of both depression and neurological deficits, restricts patients’ rehabilitation progress, impairs their quality of life, and represents a primary cause of their difficulty in reintegrating into society (4). This case report details the nursing experience of a patient with PSF managed by our team at Anqing Municipal Hospital in Anhui, China. Timely and structured nursing interventions were implemented, and the patient demonstrated significant clinical improvement. The following case report illustrates the practical application of this comprehensive approach in a clinical setting.

## Case presentation

Ethical approval has been obtained from the Institutional Review Board of Anqing Municipal Hospital due to the nature of this case report. Ethical approval number: Medical Ethics Review (2026) No.19. Written informed consent was obtained from the patient for publication of this report and any accompanying data. All personally identifiable information has been anonymized to protect patient privacy. This study is reported in accordance with the CARE Report guidelines (5).

## General data

A 71-year-old male patient was transferred to our department through emergency services on January 5, 2025. The chief complaint was sudden-onset left-sided limb weakness and slurred speech lasting for 4 hours. Past medical history included a 10-year history of hypertension, managed with regular medication but sub-optimally controlled (blood pressure ranging 140-160/90-100 mmHg), and a 5-year history of type 2 diabetes mellitus treated with oral hypoglycemic agents. Admission blood glucose was 11.2 mmol/L.

On admission, the National Institutes of Health Stroke Scale (NIHSS) score was 8, primarily due to facial palsy, left upper and lower limb motor deficits, and dysarthria. Emergency cranial magnetic resonance imaging revealed an acute cerebral infarction in the right basal ganglia region. Physical examination revealed a temperature of 36.5°C, a pulse rate of 78 beats per minute, a respiration rate of 19 breaths per minute, and a blood pressure of 155/92 mmHg. Cognitive assessment using the Mini-Mental State Examination (MMSE) yielded a score of 24, indicating mild attention deficits, short-term memory impairments, and executive function deficits.

Laboratory tests revealed the following: Complete Blood Count indicated a white blood cell count of 7.5 × 10⁹/L, hemoglobin level of 132 g/L, and platelet count of 215 × 10⁹/L. Fasting blood glucose was measured at 9.2 mmol/L, with a glycated hemoglobin level of 7.8%. The lipid profile showed total cholesterol at 5.6 mmol/L, triglycerides at 2.3 mmol/L, and low-density lipoprotein cholesterol at 3.8 mmol/L. Liver and kidney function tests, along with electrolytes and coagulation parameters, displayed no significant abnormalities. The patient had smoked for 30 years (approximately 20 cigarettes per day) and consumed alcohol occasionally. The patient holds a bachelor's degree. Family history and genetic factors reveal that all three of the patient's brothers have a history of hypertension, and the patient's spouse passed away from a stroke at the age of 65, indicating a familial predisposition to cerebrovascular diseases. There is no known family history of other genetic disorders. Psychosocial factors include the patient's prior engagement in manual labor, prolonged periods of living alone, and limited social interaction. Characterized by introversion and a strong competitive nature, the patient felt burdened by concerns about potentially imposing a negative impact on their family following illness, which led to significant economic and psychological stress, compounded by a lack of social support.

The neurological examination indicated that the patient was conscious but exhibited dysarthria; the left nasolabial groove was shallow, and the mouth corner deviated to the right. Muscle strength assessment revealed proximal left upper limb strength of Grade 3, distal left upper limb strength of Grade 2+, and left lower limb strength of Grade 4. The patient could transfer from bed to chair under supervision and ambulate short distances with minimal assistance using a walker. The patient exhibited diminished superficial sensation on the left side, while deep sensation remained intact. The tendon reflexes were hyperactive bilaterally (left ++, right ++), with no pathological signs elicited, and poor coordination of motor skills was noted.

The treatment regimen consisted of enteric-coated aspirin 100 mg daily to inhibit platelet aggregation; atorvastatin calcium 20 mg nightly for lipid regulation and plaque stabilization; management of blood pressure and blood glucose levels; and neuroprotective therapy.

For the patient exhibiting reduced muscle strength in the left limb and weakness in distal muscles, active-passive exercises and resistance training were performed to improve limb muscle strength and coordination and to prevent muscular atrophy and joint contractures, along with articulation training for speech improvement. Progressive balance training and walker-assisted ambulation were arranged according to physical condition. Rehabilitation was delivered 5 days per week during hospitalization, including 5 hours of physical therapy, 3 hours of occupational therapy, and 2 hours of speech therapy, with gradual load training as the core to hierarchically optimize motor control, daily performance, and language function.

**Figure 1:**
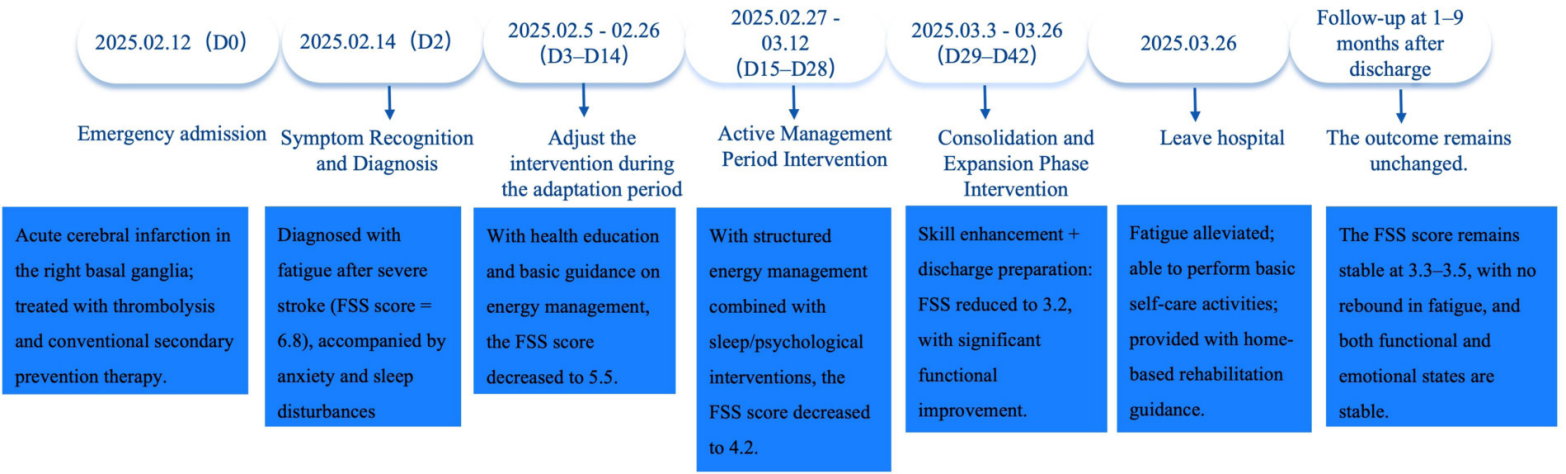
Case progress timeline.

During routine assessment, nursing staff noted that the patient's fatigue level was disproportionate to the neurological deficits. The patient reported: “I’m told my physical recovery is progressing, but I feel utterly drained from the moment I wake up. A ten-minute therapy session feels like running a marathon - afterwards, I experience palpitations, break out in a cold sweat, and cannot do anything for the next hour or two.” Family members observed, “He has become very lazy and unwilling to move. He used to be highly motivated, but now he often says, Forget it, I’ll do it tomorrow.” This profound fatigue significantly interfered with his rehabilitation, leading to poor concentration during sessions, markedly reduced exercise tolerance, and, consequently, slow progress in training. The patient also expressed anxiety and pessimism about the future, stating, “Am I just useless now?” (Figure 1).

## Multidimensional specialized assessment

To comprehensively and quantitatively evaluate the patient’s PSF and related issues, a structured assessment was completed within 48 hours of admission—details in Table 1.

**Table 1:** Multidimensional assessment results for poststroke fatigue.

Evaluation dimensions	Scale of use/Assessment method	Assessment result	Core performance/Diagnostic conclusion
Fatigue assessment	Fatigue Scale (FS), Fatigue Impact Scale Dimensional Version (FIS-D), Semi-structured Interview	The FS score averaged 6.8; the total FIS-D score was 98.	1. Meets the diagnostic criteria for post-stroke fatigue;
			2. Morning heaviness (dynamic fatigue), mild exacerbation of physical and mental exertion (exertional fatigue);
			3. Partial relief with rest, fatigue significantly influenced by emotional state and sleep quality;
			4. Severe fatigue, fatigue exerting major impact on multiple life domains.
Sleep assessment	Pittsburgh Sleep Quality Index (PSQI), patient-reported	The total PSQI score is 18.	1. Severe sleep disorders, including difficulty falling asleep, fragmented sleep, early awakening, and daytime dysfunction;
			2. Persistent nighttime worry about the condition with actual sleep duration of less than 4 hours.
Psychological assessment	Hospital Anxiety and Depression Scale (HADS), Stroke-Specific Self-Efficacy Scale	The HADS anxiety subscale scored 12 points, the depression subscale scored 9 points; the self-efficacy subscale score was low.	1. Moderate anxiety and mild depressive symptoms;
			2. Insufficient confidence in rehabilitation activities and overcoming difficulties, with low self-efficacy
Nutrition	Nutrition Risk Screening 2002 (NRS 2002)	The score is 3 points	Nutritional risk exists
	Numerical Rating Scale for Pain (NRS)	Scored 0 points	No pain symptoms
	Modified Barthel Index (MBI)	The score is 65 points	partial self-help

## Nursing interventions

### Diagnosis of PSF

**Figure 2:**
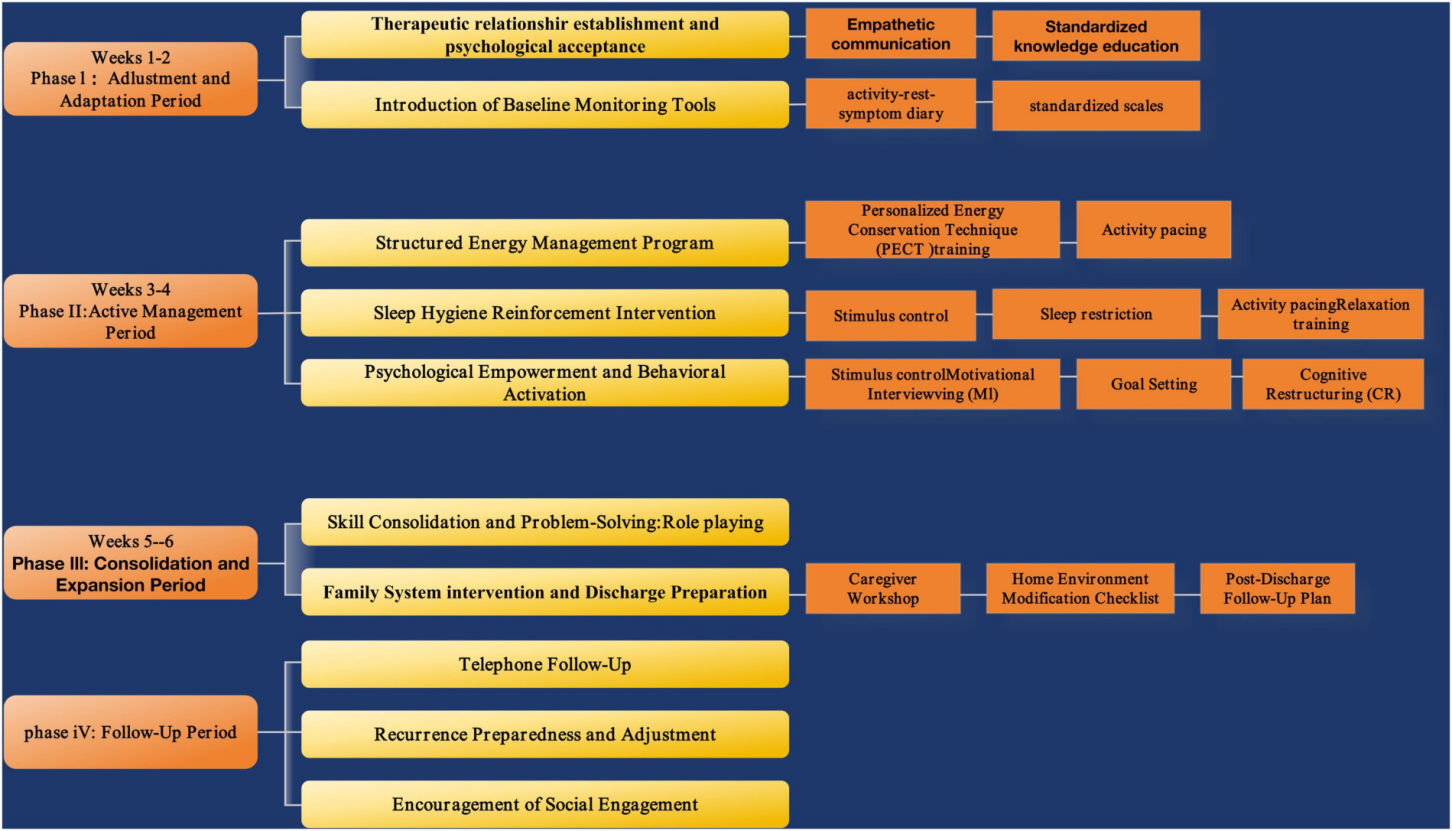
Flow chart of the four-phase intervention plan.

According to the Canadian Stroke Best Practice Recommendations: Mood, Cognition and Fatigue following Stroke (1), persistent fatigue occurring within 48 hours after stroke onset that does not alleviate with rest shows no direct correlation between fatigue severity and neurological deficits (NIHSS score of 8) or depressive symptoms (Figure 1). A Fatigue Scale (FSS) score of 6.8 and a Fatigue Impact Scale Dimensional Version (FIS-D) score of 98 meet the diagnostic criteria for severe PSF. After excluding secondary causes of fatigue such as infection, anemia, thyroid dysfunction, electrolyte disturbances, cardiopulmonary insufficiency, or adverse drug reactions, the condition is confirmed as severe PSF. Currently, there are no specific medications for treating PSF; clinical management primarily relies on non-pharmacological interventions, while medications are used solely to control underlying conditions and associated symptoms. Prior to diagnosing severe fatigue following stroke in this case, all other potential causes of fatigue were systematically ruled out: there was no evidence of adverse drug reactions; thyroid function and cortisol levels were normal, ruling out endocrine abnormalities; fatigue onset preceded depression and was independent of emotional factors, excluding a purely depressive origin; hemoglobin levels were normal, excluding anemia; and there were no signs of snoring or hypoxia, ruling out sleep apnea. In conclusion, the fatigue was attributed to PSF. This phase targeted three core domains contributing to PSF: energy expenditure patterns, sleep dysfunction, and psychological barriers. This Case Report applied a combined theoretical model of holistic nursing and empowerment nursing to conduct a six-week intervention, divided into three phases: adjustment and adaptation (weeks 1-2), active management (weeks 3-4), and consolidation and expansion (weeks 5-6). Follow-ups were conducted at 1, 3, and 6 months after discharge (Figure 2).

## Phase I: adjustment and adaptation period (weeks 1-2)

Comprehensive Assessment, Alliance Building, and Educational Foundation. The focus of this phase was to establish a mutually trusting nurse-patient relationship, educate the patient and family about PSF, and initiate baseline monitoring.

### Therapeutic relationship establishment and psychological acceptance

Empathetic communication is an interactive process co-constructed by the patient and the provider (6, 7). The initial interview lasts 60 minutes. During the initial nursing interview, the nurse began by stating, “I understand you are feeling extremely fatigued, and even rest does not seem to help. This must be very frustrating,” thereby validating the patient's experience. The nurse clearly explained, “The exhaustion you are experiencing is medically termed PSF. It is a common and genuine symptom following brain injury, much like physical weakness. It is neither your fault nor a sign of laziness.”

Standardized knowledge education was provided through an illustrated PSF Patient Handbook, which detailed the pathogenesis of PSF, its distinguishing characteristics from ordinary fatigue, common triggers, and management strategies. The nurse emphasized, “Managing fatigue is a crucial component of rehabilitation, just as important as physical exercise.”

### Introduction of baseline monitoring tools

An activity-rest-symptom diary was introduced, where the patient and family were instructed to maintain a simplified daily log collaboratively. This included recording wake/sleep times, specific activities performed (e.g., “30 minutes of physical therapy,” “self-feeding”), fatigue levels before and after activities (on a 0-10 scale), mood states, and the presence or absence of naps. The purpose was to help identify the patient's personal “energy peaks,” “energy troughs,” and potential sources of fatigue.

Additionally, standardized scales were administered periodically for objective quantitative assessment: the FSS, Pittsburgh Sleep Quality Index (PSQI), and the mood subscales of the Hospital Anxiety and Depression Scale (HADS) were re-evaluated weekly to establish a baseline of objective data.

### Phase outcomes

The patient's mean FSS score decreased from 6.8 to 5.5, with mild alleviation of daytime heaviness; adherence to rehabilitation training improved, allowing sustained performance for 10–15 minutes; the PSQI score dropped to 15, indicating slight improvement in sleep difficulties; HADS scores showed a reduction in anxiety (10 points) and depression (7 points), reflecting initial relief of negative emotions Conditions for moving to the next stage: A decrease of ≥15% in the FSS score from the baseline at stage entry, coupled with the ability to successfully complete 10–15 minutes of rehabilitation training consecutively, along with concurrent improvements in PSQI and HADS scores, meet the criteria. In this case, the FSS score decreased from 6.8 to 5.5, representing a 19.1% reduction, thereby allowing progression to Phase II.

## Phase II: active management period (weeks 3-4) - implementation of core intervention techniques

The objective of this phase was to systematically apply non-pharmacological interventions to alleviate fatigue and enhance the patient’s daytime functioning.

### Structured energy management program

Personalized Energy Conservation Technique (PECT) training was implemented, which involved task breakdown, simplification, and reorganization. Energy management training should be performed once daily for 30–40 minutes each session. In collaboration with the rehabilitation therapist, morning activities of daily living (ADLs) such as washing and dressing were deconstructed. For instance, the patient was advised to perform face washing and toothbrushing while seated, and clothing was arranged in the order of use. The patient was encouraged to incorporate 30-second “micro-breaks” (e.g., deep breathing or closing the eyes) during ADLs.

Activity pacing, a core skill, was taught by the nurse using the “plan first, execute later” principle and an “activity-rest alternation” approach. For example, a two-hour morning physical therapy session was divided into two 15-minute segments with a mandatory 10-minute rest period in between (timed using a timer). Emphasis was placed on stopping an activity before reaching exhaustion. Assistive devices such as shower chairs, long-handled reachers, and dressing sticks were introduced. The ward environment was also adjusted to ensure frequently used items were within easy reach.

Based on the energy patterns identified in the diary from the previous two weeks, a personalized “energy budget” and daily schedule were developed. Using the diary records, the nurse and patient collaboratively planned the timing of essential but energy-intensive rehabilitation sessions (“high-energy activities”) and scheduled less demanding tasks (“low-energy activities”) for the afternoon. Scheduled rest periods were formally incorporated into the ward schedule as prescribed interventions.

### Sleep hygiene reinforcement intervention

Take once daily before bedtime, for each session lasting 15–20 minutes. Stimulus control was strictly enforced by adhering to the principle that “the bed is only for sleep.” If the patient was unable to fall asleep within 20 minutes in bed, they were instructed to get up, sit in a chair, and engage in light relaxation or reading until feeling sleepy, then return to bed.

Sleep restriction was applied based on the patient's actual average sleep duration (approximately 4 hours). A strict sleep window (e.g., 1:00 AM to 5:00 AM) was initially set. Once a consistent sleep pattern was established, bedtime was gradually delayed by 15 minutes daily based on sleep log data, aiming to improve sleep efficiency while reinforcing a healthy sleep routine.

Relaxation training was conducted daily, consisting of guided progressive muscle relaxation for 15-20 minutes, starting one hour before bedtime.

A stable sleep-wake rhythm was promoted by instructing the patient to wake up at the same time every morning, regardless of the previous night’s sleep, and to expose themselves to natural light by opening the curtains immediately upon waking.

### Psychological empowerment and behavioral activation

#### Motivational interviewing (MI)

MI has become a popular approach for relieving mood disturbances (8). To address the patient's resistance, such as reluctance to engage in exercises, MI techniques were used twice weekly for 30 minutes each session. For example, the nurse asked, “If there were a way to help you feel slightly less tired while also making your leg stronger, would you be willing to try it?” Patients often resist rehabilitation due to fears about losing independence, inability to return to work, or no longer being able to fulfill family responsibilities. By linking rehabilitation activities to the patient's personal values, we used motivation-enhancing language to stimulate intrinsic motivation.

#### Goal setting

Setting goals can enhance the effectiveness of post-stroke rehabilitation (9). Guided by the SMART principles - Specific, Measurable, Achievable, Relevant, and Time-bound - nursing theory and practice were integrated to develop personalized rehabilitation goals. Strengthening the implementation of nursing interventions helped modify the patient's behavior, thereby promoting health improvement and enhancing quality of life (10). Short-term, quantifiable micro-goals were established for the patient. For example, the weekly goal was set as: “Complete two 15-minute physical therapy sessions daily, with a 5-minute rest in between.” Upon achieving these goals, progress was recorded in a notebook, and the patient received positive reinforcement (e.g., “You followed the plan today - excellent work!”). This approach aimed to enhance the patient's self-efficacy.

#### Cognitive restructuring (CR)

CR is one method hypothesized to play a role in change across many psychotherapies and for a variety of clinical presentations (11). Negative thoughts (e.g., “I will never recover”) were identified, gently challenged, and replaced with more balanced alternatives (e.g., “I am feeling very fatigued now, but by learning to manage fatigue, I am making gradual progress”).

#### Phase outcomes

The patient's FSS average score further decreased to 4.2, with significant reduction in post-exercise fatigue; rehabilitation attendance rate exceeded 85%, and the duration of effective single-session training increased to 30 minutes; the modified Barthel Index rose to 75, indicating improved self-care ability; PSQI score declined to 12, with reduced nocturnal awakenings; HADS anxiety score was 8 and depression score was 6, reflecting marked alleviation of anxiety and depressive symptoms. Conditions for moving to the next stage: The FSS score must decrease by ≥20% from the initial stage value, with each valid training session lasting ≥30 minutes, and with simultaneous improvement in the modified Barthel Index, PSQI, and HADS scores. In this case, the FSS score decreased from 5.5 to 4.2, a 23.6% reduction, meeting the criteria for progression to phase III.

## Phase III: consolidation and expansion period (weeks 5-6) - skill internalization and integration into daily life

This phase aimed to enable the patient to master and apply self-management strategies to accomplish daily life tasks independently.

### Skill consolidation and problem-solving

Role-playing was used to simulate potential real-life scenarios, such as “What would you do if you felt exhausted after socializing with guests at home for too long?” The patient was guided to generate their own solutions (e.g., “I would mention my need for rest beforehand and excuse myself for a 10-minute break during the visit”). The patient was encouraged to view themselves as the primary manager of their fatigue within the family context. Beneficial strategies were shared with family members and could also be discussed with fellow patients.

### Family system intervention and discharge preparation

The ability of a family to engage in self-care serves as the foundation for maintaining balanced family cohesion, which refers to the emotional bond and mutual attraction among family members (12, 13).

### Caregiver workshop

One to two specialized education sessions were organized for the patient's spouse; each session lasts 45 minutes. Covering the nature of PSF, methods to assist with energy conservation techniques, how to encourage without exerting pressure, recognizing fluctuations in the patient's emotional state, and the importance of caregiver self-care (to prevent burnout).

### Home environment modification checklist

A checklist of home environment improvements was provided, including removing rugs to prevent falls, installing handrails in the bathroom, and placing a comfortable recliner in the living room.

### Post-discharge follow-up plan

A follow-up plan was developed, specifying outpatient follow-up locations and schedules, community resources, and contact protocols for recurring or worsening fatigue.

### Phase outcomes

The patient's mean FSS score decreased to 3.2, with significant reduction in self-reported heaviness and persistent fatigue; rehabilitation attendance rate exceeded 95%, and the duration of each effective training session stabilized at 60 minutes; the modified Barthel Index reached 85 points, enabling independent indoor mobility with a cane; the total PSQI score dropped to 9, indicating marked improvement in sleep quality; HADS scores for anxiety (6) and depression (5) normalized, reflecting restored emotional stability. Conditions for moving to the next stage: A reduction of ≥20% in FSS score, a single training session lasting up to 60 minutes, an improved Barthel Index score of ≥80 points, normalization of HADS emotional indicators, and acquisition of fatigue self-management skills by the patient are sufficient criteria for discharge and entry into follow-up. In this case, the FSS score decreased from 4.2 to 3.2, representing a 23.8% reduction. This meets the follow-up criteria; thus, the patient is eligible for the follow-up phase.

## Phase IV: follow-up period

Follow-ups were conducted at 1, 3, and 6 months after discharge to sustain intervention outcomes and support the patient’s long-term adaptation.

### Telephone follow-up

Follow-up calls were made at 1, 4, and 12 weeks post-discharge to assess the application of fatigue management techniques, sleep quality, emotional state, and participation in community-based rehabilitation. A brief version of the FSS was administered remotely for evaluation.

### Recurrence preparedness and adjustment

Patients were informed that fluctuations in fatigue levels are normal, particularly during illness, weather changes, or emotional distress. They were advised to review their symptom diaries and management logs to adjust their “energy budget” rather than resorting to self-blame or disengagement.

### Encouragement of social engagement

When energy levels permitted, patients were gradually encouraged to participate in safe social activities. Engagement with peer support networks was promoted to foster a sense of validation and empowerment.

Patient-centered holistic care, integrating the principles of evidence-based and precision nursing, was delivered through multidisciplinary collaboration. This approach ensured continuous, personalized, coordinated, and efficient nursing services tailored to the patient's full lifecycle needs (14). Nurses engage in various care activities to empower patients with self-health management capabilities and facilitate their involvement in disease self-management and rehabilitation training. This process is termed empowerment nursing (15).

### Follow-up outcomes

At 1 month post-discharge, the FSS score remained at 3.3; at 3 and 6 months, it stabilized around 3.5, indicating sustained improvement in fatigue symptoms. Rehabilitation habits were well maintained, enabling patients to perform daily activities independently. Sleep and emotional indicators remained stable, with excellent self-management capabilities. At 9-month outpatient follow-up: The patient's sleep quality continued to improve, with a significant reduction in difficulty falling asleep and nighttime awakenings, along with stable daytime energy levels; emotional stability was observed, with no recurrence of anxiety or depressive symptoms and enhanced self-acceptance; regular participation in community-based rehabilitation training further improved activity endurance. Evaluation using the Brief Fatigue Scale (BFS) revealed that fatigue remained mild, demonstrating consistent intervention efficacy without significant rebound effects.

## Outcomes

After a 6-week, 3-month, and 6-month structured, multidimensional intervention and post-discharge follow-up, the patient showed significant improvements across multiple key indicators. Specifically, the Fatigue Severity Scale score decreased from an average of 6.8 at admission to 3.2 at discharge and stabilized around 3.5 during the 3-month follow-up, indicating substantial relief from refractory severe fatigue. Regarding sleep and mood, the total Pittsburgh Sleep Quality Index score dropped to 9, with reduced sleep latency and fewer nocturnal awakenings; the Hospital Anxiety and Depression Scale anxiety score decreased to 6 and the depression score to 5, both within the normal range. In terms of functional activity and compliance, rehabilitation therapy attendance exceeded 95% with high-quality completion of training sessions. The modified Barthel Index reached 85 at discharge (indicating mild dependence), enabling independent indoor ambulation with a cane. Furthermore, the patient and family demonstrated knowledge of poststroke fatigue and proficiency in core self-management techniques, such as activity pacing and planned rest. The patient reported having learned to coexist with fatigue and no longer experienced fear. Throughout hospitalization and follow-up, no adverse safety events such as falls or bed falls occurred—details in Table 2.

**Table 2:** Outcomes of the structured intervention for poststroke fatigue.

Evaluation dimensions	Specific effects of intervention and follow-up
Fatigue level	The FS score averaged 6.8 at admission, decreased to 3.2 at discharge, and stabilized at approximately 3.5 during the 3-month follow-up; the patient reported significant improvement in refractory severe fatigue.
Sleep and Emotion	The total PSQI score decreased to 9, with reduced sleep onset time and fewer nocturnal awakenings; the HADS anxiety score decreased to 6 and the depression score decreased to 5, both within the normal range.
Functional Activities and Compliance	The attendance rate for rehabilitation therapy exceeded 95%, with high-quality training completion; the modified Barthel Index at discharge reached 85 points (mild dependence), enabling independent ambulation indoors with the aid of a cane.
Knowledge and Self Management	Patients and their families can describe knowledge related to Post-Surgical Fatigue (PSF), demonstrating proficiency in core techniques such as activity rhythm control and planned rest; patients report having learned to coexist with fatigue and no longer experience fear.
Safety outcome	No adverse safety events such as falls or bed falls occurred during hospitalization and follow-up.

## Discussion

The success of this intervention can be attributed to several foundational principles: early recognition, holistic assessment, and patient empowerment. By using standardized tools such as the FSS, PSQI, and HADS at admission, the nursing team moved beyond subjective impressions to quantify the severity and multifaceted impact of PSF objectively. This data-driven approach facilitated the establishment of a clear baseline. It justified the allocation of targeted resources, a critical step often missing in routine stroke care, where fatigue may be misattributed to depression or poor motivation (16).

Our results strongly support and extend existing evidence on the efficacy of core components within such interventions. The observed benefits of personalized Energy Conservation Techniques (ECT) and activity pacing align with systematic reviews highlighting their role in breaking the “boom-bust” cycle of overexertion and crash, thereby improving daily functioning. By translating these principles into a collaborative “energy budget” and integrating them into specific activities of daily living (ADLs), we moved beyond generic advice to foster practical, sustainable self-management skills. Similarly, the positive outcomes associated with motivational interviewing (MI) and SMART goal-setting corroborate research emphasizing the need to address the motivational deficits and low self-efficacy inherent in PSF. The mechanism likely involves enhancing intrinsic motivation by linking manageable behavioral tasks (e.g., completing a shortened therapy session) to the patient's core values (e.g., regaining independence), thereby promoting a sense of autonomy and competence, which are central to behavioral activation.

A pivotal, yet often under-implemented, aspect of our intervention was the concurrent, rigorous focus on sleep hygiene based on principles of Cognitive Behavioral Therapy for Insomnia (CBT-I) (17). The significant improvement in sleep quality (reduction in PSQI score) likely created a positive neurophysiological feedback loop. By addressing sleep fragmentation—a known exacerbator of central fatigue and cognitive dysfunction—we may have enhanced diurnal arousal regulation and increased the patient's capacity to engage with and benefit from daytime energy management and rehabilitation activities. This integrated approach to sleep and fatigue management represents a critical synergy often missed in fragmented care models.

This case offers several unique insights for clinical practice. First, it underscores the indispensable role of the nurse as a case manager and coordinator in PSF care (18). PSF's etiology is multifactorial, straddling neurological, physiological, cognitive, and psychological domains. The nurse's role in conducting holistic assessments, synthesizing input from rehabilitation, medical, and psychological perspectives, and ensuring consistent messaging across all touchpoints was fundamental to the intervention's coherence and success (19). Second, we highlight the transformative impact of systematic caregiver education. By demystifying PSF for the patient's spouse, we converted a potential source of misunderstanding (“laziness”) into a knowledgeable ally, thereby strengthening the patient's social support system - a key determinant of long-term adherence and outcomes (20). This family-system approach is crucial for generalizing learned strategies from the clinical setting to the home environment.

This study has several limitations. As a single-case report, the findings lack generalizability. The patient's improvement may be partially attributed to natural recovery and conventional rehabilitation therapy. The patient's relatively high educational level and strong family support may have contributed to the positive outcomes and may not be representative of all stroke survivors. In addition, the intensive, multi-component 6-week intervention requires substantial nursing time and expertise, which may hinder its widespread application in resource-limited settings. Finally, potential placebo and non-specific therapeutic effects—such as supportive interaction, regular follow-up, and patient expectations—cannot be ruled out, and the absence of a control group limits causal inference, making it difficult to distinguish intervention-specific benefits from natural recovery or routine rehabilitation.

Based on our experience, we propose concrete steps for practice and research. Clinically, we advocate for the routine integration of a brief, validated PSF screening tool (e.g., the Fatigue Severity Scale) into the standard nursing assessment for all stroke patients. Those screening positive should then receive a comprehensive evaluation and be offered structured management programs. To enhance scalability, future work could develop and test streamlined, stepped-care models or digital health applications that deliver core components of education, activity pacing, and sleep hygiene with less intensive professional input. For research, robustly designed randomized controlled trials are needed to validate the efficacy of this specific multimodal protocol compared with standard care and to identify which patient subgroups benefit most from which combination of interventions (21).

In conclusion, PSF is a complex but treatable barrier to stroke recovery. This case illustrates that a coordinated, evidence-based approach—encompassing structured energy management, sleep optimization, motivational support, and family engagement—can empower patients, alleviate fatigue, and improve functional outcomes.

## Patient perspective

The patient expressed high overall satisfaction with the treatment, noting that the medical communication was patient and the procedures were smooth, effectively alleviating initial diagnostic anxiety. The rehabilitation plan was progressive, accompanied by clear home guidance to facilitate long-term adherence, and resulted in significant improvements in both heel pain and functional mobility. Health education helped the patient build confidence in managing their disease, resulting in a positive overall healthcare experience.

## Conclusion

This detailed case report illustrates the potential of a structured, nurse-coordinated, biopsychosocial approach to mitigate severe PSF. It underscores the necessity of routine PSF screening post-stroke and calls for further controlled studies to evaluate the efficacy of such integrated protocols.

## Data Availability

The raw data supporting the conclusions of this article will be made available by the authors, without undue reservation.
